# Volumetric Absorptive Microsampling in Therapeutic Drug Monitoring of Immunosuppressive Drugs—From Sampling and Analytical Issues to Clinical Application

**DOI:** 10.3390/ijms24010681

**Published:** 2022-12-30

**Authors:** Arkadiusz Kocur, Tomasz Pawiński

**Affiliations:** Department of Drug Chemistry, Medical University of Warsaw, Banacha 1, 02-097 Warsaw, Poland

**Keywords:** microsampling, therapeutic drug monitoring, personalized therapy, immunosuppressants, VAMS, transplantation

## Abstract

Miniaturisation and simplification are novel approaches in clinical bioanalysis, especially in therapeutic drug monitoring (TDM). These contemporary trends are related to the sampling, pre-treatment, and analysis of biological fluids. Currently, dried blood spot (DBS), one of the most popular microsampling techniques, is feasible and inexpensive. However, obtaining reliable results with sample homogeneity and volume variability is difficult. Volumetric Absorptive Microsampling (VAMS) has recently enabled the accurate and precise collection of a fixed blood volume. It reduced the hematocrit effect, improved volumetric accuracy, and generated results correlating with the dose and drug exposure from wet blood. This review focuses on VAMS-Mitra™ devices, which have become increasingly important since 2014, mainly for TDM and toxicology studies. First, the current literature has been reviewed based on immunosuppressants and their determination in samples obtained using Mitra™. Second, the critical points, weaknesses, and strengths have been characterized in contrast to classic venipuncture and other microsampling methods. Finally, we indicate the points of attention according to the perspective of Mitra™ as well as its usefulness in clinical practice. VAMS is currently state-of-the-art in microsampling and seems to be a good instrument for improving adherence to immunosuppressive therapy, especially in the pediatric population.

## 1. Therapeutic Drug Monitoring of Immunosuppressive Agents

Pharmacotherapy with some drugs, especially those characterized by a narrow therapeutic index (NTIDs), requires the determination of their concentrations in body fluids to avoid under- or overdosing [1]. Therapeutic drug monitoring (TDM) is necessary during therapy with widely used antiepileptics (carbamazepine, valproic acid, and phenytoin), antipsychotics (clozapine and lithium salts), aminoglycosides (gentamicin), digoxin, and rarely antifungal, antiviral, and biological drugs. Immunosuppressants, including tacrolimus (TAC), cyclosporine (CSA), sirolimus (SIR), everolimus (EVE), and mycophenolic acid (MPA), are among the most frequently monitored drugs in the TDM process. Additionally, some studies have exceptionally justified the monitoring of MPA, such as side effects, unpredictable pharmacokinetics in personalized therapy, and detection of adherence to the therapy level [1,2]. A few main factors determine the necessity of TDM of immunosuppressants: narrow therapeutic window and targeted concentration range, severe consequences of missing therapeutic drugs (drug toxicity/graft dysfunction), high dose/exposure ratio, and critical adherence to the therapeutic regimen [3,4].

TAC is a calcineurin inhibitor, the cornerstone of immunosuppressive therapy after solid organ transplantation (SOT) in adult and pediatric transplant recipients. Due to unpredictable intraindividual pharmacokinetics (PK) and high inter-individual variability, improvements in TDM, including sampling, are still needed. TAC’s target steady-state concentration range varies from 5 to 20 ng/mL (during the initiation of therapy, from 2 ng/mL) in adults and children. TAC binds strongly to red blood cells (RBC) at approximately 85%; therefore, whole blood is a suitable matrix for drug determination. The trough concentration (C_trough_) is a routine PK parameter monitored during the TDM of TAC. The stability of TAC as an analyte at ambient temperature (room temperature, RT) was the highest among all the immunosuppressive drugs (14 days). Liquid chromatography-tandem mass spectrometry (LC-MS/MS) is the most popular method for TAC determination because over 50% of TDM laboratories have declared this method a routine TAC quantification protocol. Rarely, immunochemical assays (IA) are used to monitor TAC, namely, the enzyme multiplied immunoassay technique (EMIT), antibody-conjugated magnetic immunoassay (ACMIA), chemiluminescent microparticle immunoassay (CMIA), electrochemiluminescence immunoassay (ECLIA), and quantitative microsphere system (QMS). Limited sensitivity and selectivity are regarded as fundamental flaws of IAs. The International Association of Therapeutic Drug Monitoring and Clinical Toxicology (IATDMCT) recommends LC-MS/MS as a reference for immunosuppressant determination because the superior specificity and selectivity of this technique allows for a balance between dosages in the therapeutic range during immunosuppressive pharmacotherapy [1,4,5,6].

CSA is also used for treatment after SOT and is interchangeable with TAC. The mechanism of action is based on the indirect inhibition of calcineurin via direct binding to the cyclophilin complex [7]. LC-MS/MS is the gold standard for CSA determination, but IAs are also used in this case, such as EMIT, ACMIA, CMIA, ECLIA, and cloned enzyme donor immunoassay (CEDIA). The estimated concentration range was 50–350 ng/mL and 480–2000 ng/mL for C_0_ and C_2_ (concentration measured before dosing and after 2 h of drug administration) PK parameters, respectively. The most suitable matrix for CSA determination is whole blood because this drug is partly bound to RBC (~41–58%). The stability of the CSA in the samples was experimentally determined within seven days [1,4].

SIR and EVE are mTOR kinase inhibitors in whole blood because of their strong binding to RBC (95% and 75%, respectively). The stability values in RT were similar to those in CSA for both drugs. The estimated concentration ranges oscillate at 3–20 ng/mL and 3–15 ng/mL for SIR and EVE, respectively [1,8]. LC-MS/MS is also recommended as a reference method for SIR and EVE determination by IATDMCT. However, IAs (CMIA, AC-MIA, EMIT for SIR, and QMS for EVE) have also been performed, particularly in smaller TDM laboratories [1,8].

An Inosine-dehydrogenase-monophosphate inhibitor (IMPDH), MPA, administered as a prodrug, mycophenolate mofetil (MMF), or the sodium salt of MPA, should be determined in different matrices. In total, 99.9% of the dose is distributed in the plasma; therefore, EDTA-plasma is preferentially recommended as a suitable target for the TDM of MPA. The typical concentration observed in the TDM samples varied between the 1–4 μg/mL concentration range. Relatively high values allow the determination of MPA using high-performance liquid chromatography with ultraviolet detection (HPLC/UV) and LC-MS/MS. Guidelines recommend both techniques as the reference gold standard for MPA quantification. Typical IAs used for MPA determination are similar to previously reported analytes (EMIT and CEDIA); however, the particle-enhanced turbidimetric inhibition immunoassay (PETINIA) and IMPDH-based enzyme inhibition assays are characteristic only for MPA quantification [1,2].

As a systematic approach, conventional venipuncture is the gold standard in clinical practice for toxicology, drug monitoring, and morbidity diagnosis. During this procedure, a higher volume of whole blood is collected (>0.1–5 mL) in relation to the microsampling methods. The main disadvantage of phlebotomy is its invasiveness. Additionally, reliable sample examination is complicated by the unique requirements for storage and delivery to the diagnostic laboratory. Medical staff must collect invasive samples because of the appropriate high quality of the sample. Venous blood is collected from the elbow, arm, and forearm, whereas capillary blood may be collected from the earlobe, forearm, heel, palm, and fingertip. Fingerpricking is noninvasive, painless, and more straightforward than classic venipuncture. It should be noted that the results of microsampling methods should be evaluated using the reference venipuncture method; however, in most cases, equivalence with or without correction factors has been demonstrated [9,10].

TDM of immunosuppressants requires frequent blood sampling to determine overall drug exposure and dose adjustment. The most critical part of the analytical process is selecting a suitable sampling method and sample preparation. Because of the prevalence of extremely sensitive and accurate analytical techniques, efforts are being made to minimize the amount of material collected for research while maintaining appropriate sensitivity. Microsampling is a noninvasive procedure with small body fluid volumes, usually less than 50 μL [11,12]. In clinical chemistry, nonvolumetric methods, such as capillary microsampling (CMS) and dried blood spots (DBS), have well-established positions. Characteristic problems for nonvolumetric techniques, especially DBS, include the volcano effect, the hematocrit effect, and limited sample homogeneity. These problems and the desire to reduce the amount of material collected for research have contributed to the development of new volumetric absorption microsampling (VAMS) techniques that have gained importance for monitoring therapy in recent years [10]. Microsampling has numerous advantages, which make it more attractive [11,12,13], namely, even 200 times less collected whole blood volume than classic phlebotomy, less invasiveness for patients, especially children, home-based sampling, and simple sample collection, storage, and delivery/shipment. The biological risk is minimized because of small biological fluid volumes and self-sampling with safe one-use lancets. However, limited data on the clinical application of these devices, higher unit prices, and special analytical equipment with higher sensitivity (LC-MS/MS system) may be treated as limitations of microsampling [12].

In that review, particular focus was placed on the VAMS—Mitra™ utility in immunosuppressant TDM. Additionally, other volumetric devices have been described based on immunosuppressive agent monitoring.

To our knowledge, this is the first comprehensive analytical and clinical review of the utility of VAMS devices for immunosuppressant monitoring.

## 2. Volumetric Absorptive Microsampling (VAMS) in the Current Literature (2014–2022)

Volumetric absorptive microsampling (VAMS), introduced by Neotheryx LLC (Torrance, CA, USA), was used to collect blood samples at the end of 2016. It is a high-precision volumetric technology named Mitra^™^, developed by Dennif and Spooner in 2014 [13,14,15]. More than 100 different investigations focusing on the VAMS-Mitra™ device have been performed. Keeping up with the producer, the Mitra™ device is characterized by an uncomplicated design; namely, the microsampler contains the tip set on the sampler body with ribs, a barrel, and the distal end. The sampler tip is built using a hydrophilic porous polymer, which rapidly wicks biological fluids, such as whole blood, serum, urine, breast milk, spinal liquid, and saliva. On the market, three volumes of tips are available: 10 μL, 20 μL, and 30 μL.

The first is dedicated to small molecules, the second to biochemical analysis, and the third is designed for genetic and molecular biology methods. A total of 10 μL is commonly used for drug monitoring in LC-MS/MS analyses, 20 μL tips are required for larger molecules (e.g., protein-based biomarkers or immunoassay methods), and 30 μL is used in molecular diagnostics (for example, RNA/DNA extraction) [12,15].

Generally, larger volumes of tips contain larger pores. Therefore, larger molecules may be analyzed in this type of VAMS. For every pack of VAMS, the analytical certificate is added, with information about the exact volume of samplers in that lot (with RSD < 4%) [15]. The sampler body had ribs that prevented the samples from encountering the walls of the extraction tube. The barrel is used for the labeled sampler, whereas the distal end is suitable for standard automatic hand pipettes smaller than 200 μL volume [13,15]. The VAMS tips absorbed whole capillary blood within a few seconds (2–4 s range) and dried rapidly at room temperature (RT) for a maximum of 2 h. The drying process increases the stability level of the sample, and transport of that sample is more accessible because of logistic reasons. The tip may or may not be removed from the handler before the extraction [13,15]. For example, the body fluid may be collected in situ from the finger previously disinfected and punctured by a classic lancet. The first blood drop should be discarded, and the sampler may collect the second capillary drop. In the case of blood, where the fluid is colored, the assessment of the wick grade is more accessible than in other fluids (which are colorless or the color is not significant), and the sample is successfully collected when the entire tip is red. The collection process appeared to be extremely easy and could be performed without special training, similar to measuring blood sugar levels. The Mitra™ device is classified by the FDA as class 1, with an accurate and precise collection of a fixed blood volume [11,15].

The sampler may be left for drying and storage in a 96-placed autorack, 1–4 placed clamshells, or 2-placed cartridges. Samples were placed in zip bags in the dark with desiccant and delivered to the laboratory via classic mail. VAMS can be analyzed manually step-by-step with the possibility of automatization [13,14,15,16].

**Figure 1:**
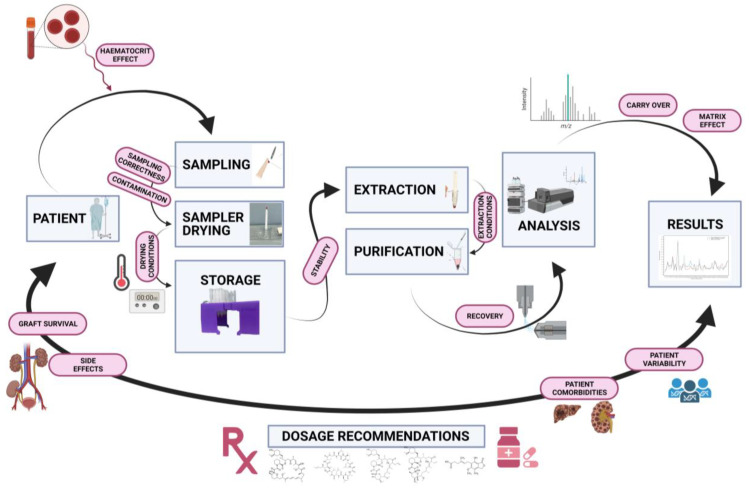
Preanalytical, analytical, and clinical process summary with the main critical points. Created with BioRender.com under publishing rights.

The VAMS sample method from the patient to the medical laboratory and the results obtained using the classic critical point are presented in Figure 1. In the following section, all steps are described according to the current literature and therapeutic drug monitoring of immunosuppressive agents, including clinical outcomes.

### 2.1. Pre-Sampling

In the case of VAMS, the tip with adsorbed blood may or may not be removed by the plastic handler. The unique patient label bar code could be present during the preparation process until the end of extraction. Ideally, the sampler should be incorporated with IS before sample collection, but this approach may be problematic in most studies because of stability reasons and potential home-sampling destiny. All devices were equipped with barcode labels, allowing for sample confusion in potential process automation.

When using microsampling as a potential device for self-sampling by inexperienced patients, extensive training by medical staff is greatly needed, as well as regular revision during follow-up visits. Explicit sampling instruction, tutorial videos, and other supplementary materials are excellent addendums for pre-sampling process correction [12,13,14,15].

### 2.2. Sampling

Denniff and Spooner thoroughly studied the potential difficulties encountered during sampling by VAMS [12,16,17]. Sampling from a greasy finger caused a 10% lower wicking by the tip concerning the disinfected finger. Accidentally dropping the sampler into the floor caused approximately 3% analyte loss [12,16]. The physical contact of the loaded tip with other materials, such as an unloaded tip and paper, caused 10 and 19% of analyte loss, respectively [12,16]. However, the above study focused on simple analytes such as caffeine, paracetamol, and midazolam, but different scenarios should be performed during method development and validation in the case of immunosuppressants [17]. Because of the variable volumes of tips, the total time of filling by blood may be different. The potential problems indicated during sampling are shown in the figure. Following manufacturer guidelines, the tip should be dry under ambient conditions, especially at room temperature (RT) and relative humidity of 55% for one hour, but no longer than 24 h [15]. In microsampling studies of immunosuppressant agents, different approaches to drying times have been observed (Table 1) [16,17].

Some studies have reported that 24 h is a suitable time for drying VAMS samplers under ambient conditions. In our study about TAC [18], a reduction in the drying time to 1 h ensures a high recovery of TAC and no significant differences between the 2 h and 1 h period of VAMS drying. Vethe et al. [19] reported that 3 h of VAMS drying is satisfactory; however, Kita et al. [20,21] and Koster et al. [22] reported that the period of drying VAMS may be reduced to 2 h with no significant differences in TAC extraction accuracy.

In a comparison study of DBS and VAMS, all samples for DBS were collected with acceptable visual quality by a phlebotomist and by 94.1% of transplant patients. In the case of VAMS, samples for the simultaneous determination of TAC and MPA were collected with 95.2% and 70.6% accuracy for the phlebotomist and self, respectively [22]. It does not prejudge more difficult sampling with VAMS over DBS but confirms the necessity of intensive training before VAMS self-patient sampling. The hematocrit (HCT) effect (described in Section 4.1), blood density, co-medications with anticoagulant drugs, and individual patient comorbidities may directly impact the sampling process [23,24,25].

**Table 1:** Summary of the preanalytical characteristics of immunosuppressive drug assays using VAMS microsampling techniques.

Drug Name	Matrix	Sample Volume	Storage	Drying Method	Sampling Correctness	Microsampling Device	Reference
CSA	fingerprick CB	20 μL	n.d	n.d	n.t.	Mitra™	[22]
	fingerprick CB	20 μL	zip-lock bags with desiccant (−20 °C until analysis)	RT, 2 h	n.t.	Mitra™	[23]
	fingerprick CB	20 μL	RT, ambient conditions	RT, 24 h	n.t.	Mitra™	[26]
	fingerprick CB	20 μL	zip-lock bags with desiccant (−20 °C until analysis)	RT, 2 h	n.t.	Mitra™	[27]
TAC	fingerprick CB	10 μL	RT, ambient conditions	RT, 3 h	satisfactory	Mitra™	[19]
	fingerprick CB	10 μL	RT, ambient conditions	RT, 2 h	n.t.	Mitra™	[20]
	tail prick CB	10 μL	freezing in tube	RT, 2 h	n.t.	Mitra™	[21]
	fingerprick CB	20 μL	n.d	n.d	n.t.	Mitra™	[22]
	fingerprick CB	20 μL	zip-lock bags with desiccant (−20 °C until analysis)	RT, 2 h	MS: 95.20% SS: 70.06%	Mitra™	[23]
	fingerprick CB	20 μL	RT, ambient conditions	RT, 24 h	n.t.	Mitra™	[26]
	fingerprick CB	20 μL	zip-lock bags with desiccant (−20 °C until analysis)	RT, 2 h	n.t.	Mitra™	[27]
	fingerprick CB	20 μL	zip-lock bags with desiccant (−20 °C until analysis)	RT, 24 h	67.7%	Mitra™	[28]
	fingerprick CB	10μL	RT, ambient conditions	RT, 2 h	n.t.	Mitra™	[29]
	fingerprick CB	20μL	zip-lock bags with desiccant	RT, 2 h	n.t.	Mitra™	[30]
	fingerprick CB	10 μL	4 °C, darkness	RT, 1 h	n.t.	Mitra™	[18]
	fingerprick CB	20 μL	RT, ambient conditions	RT, 24 h	n.t.	Mitra™	[31]
	fingerprick CB	10 μL	At least 24 h in a specimen bag	RT, 24 h	n.t.	HemaXis™	[32]
MPA	fingerprick CB	20 μL	n.d	n.d	n.t.	Mitra™	[22]
	fingerprick CB	20 μL	zip-lock bags with desiccant (−20 °C until analysis)	RT, 2 h	MS: 95.20% SS: 70.06%	Mitra™	[23]
	fingerprick CB	20 μL	zip-lock bags with desiccant	RT, 2 h	n.t.	Mitra™	[30]
	fingerprick CB	10 μL	RT, ambient conditions	RT, 24 h	n.t.	HemaXis™	[32]
EVE	fingerprick CB	20 μL	n.d	n.d	n.t.	Mitra™	[22]
	fingerprick CB	20 μL	zip-lock bags with desiccant (−20 °C until analysis)	RT, 2 h	n.t.	Mitra™	[23]
	fingerprick CB	20 μL	RT, ambient conditions	RT, 24 h	n.t.	Mitra™	[26]
SIR	fingerprick CB	20 μL	n.d	n.d	n.t.	Mitra™	[22]
	fingerprick CB	20 μL	zip-lock bags with desiccant (−20 °C until analysis)	RT, 2 h	n.t.	Mitra™	[23]
	fingerprick CB	20 μL	RT, ambient conditions	RT, 24 h	n.t.	Mitra™	[26]
	fingerprick CB	10 μL	n.d.	n.d.	n.t.	Mitra™	[33]
	fingerprick CB	10 or 20 μL	20 ± 5 °C, <40% humidity, zip-lock bags with desiccant	RT, n.d.	MS: 39.1% (reduced to 13.6%)	Mitra™	[34]

CSA—cyclosporine, TAC—tacrolimus, MPA—mycophenolic acid, EVE—everolimus, SIR—sirolimus, CB—capillary blood, n.t.—not tested, n.d.—no data available in the study, RT—room temperature.

### 2.3. Sample Preparation (Extraction and Purification)

The extraction process is considered one of the critical points during sample analysis. Different approaches have been observed, such as using non-organic or organic solvents or mixtures. However, Ye and Gao [24] reported that a lower elution strength characterized a mixture of organic solvents and water, and they used particulars relatively more frequently. In some protocols, organic solvents, such as acetonitrile or methanol, were used, but they may influence analyte recovery by chopping the porous tip due to protein denaturation. According to presented TAC determination study, water was used as simple and satisfactory extraction medium. The detailed characteristics of all VAMS extraction and purification protocols are presented in Table 2.

In pilot proficiency testing, it has been pointed out that in most laboratories, IS is added during extraction, and only in one case directly on the sample or after extraction [35]. Bought calibrators and QC (Quality Control) were used in more than 60% of laboratories, whereas self-made solutions were used in the others [35]. Zinc sulphate and acetonitrile were the most frequently used solvents for sample purification, whereas organic and water mixtures were used in almost all cases for the extraction protocol. Vortexing and centrifugation are the most popular mechanical techniques for sample cleaning. Gruzdys et al. [26] presented modifications according to sample cleaning, namely, the evaporation of the sample after extraction has been connected with reconstitution with organic solvents and mechanic centrifugation.

**Table 2:** Summary of sample preparation characteristics of immunosuppressive drug assays in common with microsampling techniques.

Drug Name	Microsampling Method	Extraction Solvent	Extraction Conditions	Solvent for Sample Purification	Purification Conditions	Additional Steps	Reference
CSA	Mitra™	methanol: water (with IS); (40:60, *v*/*v*%)	sonication (30 min)	methanol	vortexing (15 min, low speed, 1 min maximal speed), sonication (15 min), vortexing (the same conditions as above), centrifugation (5 min, 10,000 g), and storage at −20 °C (10 min), centrifugation (the same conditions above)	n.d.	[22]
	Mitra™	methanol (with IS); (62.5:37.5, *v*/*v*)	sonication (15 min)	methanol	sonication (15 min), centrifugation (5 min, 14,500 g)	evaporation to dry, reconstitution with the mobile phase	[23]
	Mitra™	methanol: water (with IS); (80:20, *v*/*v*)	sonication (15 min), vortexing (60 min), centrifugation (10 min, 18,403.2 g)	n.d.	vortexing (15 min), centrifugation (10 min, 18,403.2 g)	evaporation to dry, reconstitution with the mobile phase	[26]
	Mitra™	IS solution	sonication (30 min)	methanol	vortexing (15 min), sonication (15 min), centrifugation (5 min, 13,000 g), storage at −20 °C (10 min), centrifugation (the same conditions above)	n.d.	[27]
TAC	Mitra™	water	shaking (15 min)	methanol: zinc sulphate (2:1, *v*/*v*)	shaking (6 min) centrifugation (2000 g, 10 min, 4 °C)	n.d.	[19]
	Mitra™	methanol: water (1:1, *v*/*v*)	sonication (15 min)	methanol: acetonitrile (1:1, *v*/*v*)	centrifugation (13,000 g, 5–15 min, 4 °C),	n.d.	[20]
	Mitra™	methanol: water (1:1, *v*/*v*)	sonication (15 min)	methanol: acetonitrile (1:1, *v*/*v*)	centrifugation (13,000 g, 15 min, 4 °C),	n.d.	[21]
	Mitra™	methanol: water (with IS); (40:60, *v*/*v*%)	sonication (30 min)	methanol	vortexing (15 min, low speed, 1 min maximal seed), sonication (15 min), vortexing (the same conditions as above), centrifugation (10,000 g), and storage at −20 °C (10 min), centrifugation (the same conditions above)	n.d.	[22]
	Mitra™	methanol (with IS); (62.5:37.5, *v*/*v*)	sonication (15 min)	methanol	sonication (15 min), centrifugation (5 min, 14,500 g)	evaporation to dry, reconstitution with the mobile phase	[23]
	Mitra™	methanol: water (with IS); (80:20, *v*/*v*)	sonication (15 min), vortexing (60 min), centrifugation (10 min, 18,403.2 g)	n.d.	vortexing (15 min), centrifugation (10 min, 18,403.2 g)	evaporation to dry, reconstitution with the mobile phase	[26]
	Mitra™	the internal standard solution	sonication (30 min)	methanol	vortexing (15 min), sonication (15 min), centrifugation (5 min, 13,000 g), storage at −20 °C (10 min), centrifugation (the same conditions above)	n.d.	[27]
	Mitra™	methanol: water (with IS), (80:20, *v*/*v*)	sonication (30 min)	methanol and zinc sulphate solution	vortexing (15 min), sonication (15 min), vortexing (15 min), centrifugation (10,000 g, 5 min), and storage at −20 °C (10 min), centrifugation (the same conditions above)	n.d.	[28]
	Mitra™	water with IS (50:50, *v*/*v*)	shaking (15 min), sonication (10 min),	acetonitrile and zinc sulphate mixture (1:1, *v*/*v*)	centrifugation (16,260 g, 5 min, 8 °C)	salting out with ammonium sulphate	[29]
	Mitra™	50% methanol solution	sonication (10 min), vortexing (20 min)	acetonitrile and zinc sulphate mixture (1:1, *v*/*v*) with IS	shaking (10 min) centrifugation (2900 rpm, 5 min)	n.d.	[30]
	Mitra™	water	shaking (60 min)	acetonitrile and zinc sulphate mixture (1:1, *v*/*v*)	shaking (10 min) centrifugation (3500 rpm, 10 min, 4 °C)	n.d.	[18]
	Mitra™	acetonitrile: water (40:60, *v*/*v*%)	vortexing (10 min), sonication (15 min), vortexing (10 min)	acetonitrile with IS	vortexing (5 min), centrifugation (11,337 g, 5 min)	n.d.	[31]
	HemaXis™	IS solution in methanol	vortexing (15 min)	zinc sulphate solution	centrifugation (16,000 g, 5 min)	n.d.	[32]
MPA	Mitra™	methanol: water (with IS); (40:60, *v*/*v*%)	sonication (30 min)	methanol	vortexing (15 min, low speed, 1 min maximal seed), sonication (15 min), vortexing (the same conditions as above), centrifugation (10,000 g), and storage at −20 °C (10 min), centrifugation (the same conditions above)	n.d.	[22]
	Mitra™	methanol (with IS); (62.5:37.5, *v*/*v*)	sonication (15 min)	methanol	sonication (15 min), centrifugation (5 min, 14,500 g)	evaporation to dry, reconstitution with the mobile phase	[23]
	Mitra™	50% methanol solution	sonication (10 min), vortexing (20 min)	acetonitrile and zinc sulphate mixture (1:1, *v*/*v*) with IS	shaking (10 min) centrifugation (2900 rpm, 5 min)	n.d.	[30]
	HemaXis™	IS solution in methanol	vortexing (15 min)	zinc sulphate solution	centrifugation (16,000 g, 5 min)	n.d.	[32]
EVE	Mitra™	methanol: water (with IS); (40:60, *v*/*v*%)	sonication (30 min)	methanol	vortexing (15 min, low speed, 1 min maximal seed), sonication (15 min), vortexing (the same conditions as above), centrifugation (10,000 g), and storage at −20 °C (10 min), centrifugation (the same conditions above)	n.d.	[22]
	Mitra™	methanol (with IS); (62.5:37.5, *v*/*v*)	sonication (15 min)	methanol	sonication (15 min), centrifugation (5 min, 14,500 g)	evaporation to dry, reconstitution with the mobile phase	[23]
	Mitra™	methanol: water (with IS); (80:20, *v*/*v*)	sonication (15 min), vortexing (60 min), centrifugation (10 min, 18,403.2 g)	n.d.	vortexing (15 min), centrifugation (10 min, 18,403.2 g)	evaporation to dry, reconstitution with the mobile phase	[26]
SIR	Mitra™	methanol: water (with IS); (40:60, *v*/*v*%)	sonication (30 min)	methanol	vortexing (15 min, low speed, 1 min maximal seed), sonication (15 min), vortexing (the same conditions as above), centrifugation (10,000 g), and storage at −20 °C (10 min), centrifugation (the same conditions above)	n.d.	[22]
	Mitra™	methanol (with IS); (62.5:37.5, *v*/*v*)	sonication (15 min)	methanol	sonication (15 min), centrifugation (5 min, 14,500 g)	evaporation to dry, reconstitution with the mobile phase	[23]
	Mitra™	methanol: water (with IS); (80:20, *v*/*v*)	sonication (15 min), vortexing (60 min), centrifugation (10 min, 18,403.2 g)	n.d.	vortexing (15 min), centrifugation (10 min, 18,403.2 g)	evaporation to dry, reconstitution with the mobile phase	[26]
	Mitra™	methanol with IS	sonication (15 min)	n.d.	vortexing, centrifugation (10 min, 15,000 g)	evaporation to dry, reconstitution with the mobile phase	[33]
	Mitra™	water with IS (20:1, *v*/*v*)	sonication (20 min)	LLE with tert-butyl-methyl-ether	freezing (−60 °C)	evaporation to dry, reconstitution with the mobile phase	[34]

CSA—cyclosporine, TAC—tacrolimus, MPA—mycophenolic acid, EVE—everolimus, SIR—sirolimus, n.d.—no data available in the study, IS—internal standard, LLE—liquid–liquid extraction.

### 2.4. Analytical Assay Characteristics

Chromatographic methods, especially LC-MS/MS, are considered the gold standard for small molecule determination in limited body fluid volumes, especially immunosuppressants and other NTIDs. These methods are primarily based on electrospray ionization (ESI) positive mode with a triple quadrupole detector. Paniagua-Gonzales compared ESI and unispray (US) devices for immunosuppressive MS assays [23].

Preferentially, the validation of the method according to a few analytes is currently observed—TAC, CSA, MPA, EVE, SIR, and PRE may be developed in a single run. Creatinine level is an additional biomarker for monitoring graft function in patients after renal transplantation. Using VAMS, the metabolite levels could also be measured. In the study by Scuderi et al., the MPAG level (mycophenolic acid glucuronide) was quantified. Although different MRM pairs (quantitative and control) for MPA and MPAG were established, the effect of metabolite fragmentation was negligible [30]. The selected apparatus and chromatographic parameters for all prescribed methods are presented in Table 3. Only Gruzdys et al. presented limited data on chromatographic and MS detector working conditions during analysis [26].

### 2.5. Clinical Outcome

Several factors may have influenced the patients’ clinical picture, including comorbidities, co-medications, and intertemporal PK variability. The side effects of pharmacotherapy, adherence to therapy, and graft conditions are individual for every patient may strongly influence clinical decisions regarding dosage recommendations.

Paniagua-Gonzalez et al. reported that the VAMS method for TAC and MPA determination was equivalent to the wet blood method reference, but correction factors were introduced. Mean differences were acceptable for both analytes based on Bland–Altman plots (2.20% and 2.04% bias for TAC and MPA, respectively) [23]. In a study by Scuderi et al., systematic bias was identified for TAC, and the correction of its values showed no significant differences. No statistical differences were observed between MPA and PRE in this case [30]. In the case of the newest analytical method for EVE determination, no systematic bias or HCT effect was observed, and a strong correlation between wet blood and VAMS was evaluated [33,34]. Zwart et al. deduced that the VAMS technique was generally worse than DBS, but it seems to be an attractive alternative in certain situations (i.e., for graft monitoring) [27,32]. In a study by Gruzdys et al. on four immunosuppressive agents, the highest proportional bias was observed only in the case of TAC (15%, intercept 0.3 ng/mL) [26]. The microsampling method collection and extraction showed statistically calculated CV% < 10%, excluding SIR. In two studies by Kita, the methods of tacrolimus determination were suitable for PK monitoring of TAC based on a study performed on rats [20,21]. For clinical use of this method, a correction formula based on Deming regression was introduced because of the relatively high impact of HCT during EVE determination. Vethe et al. deduced that two tips should be collected simultaneously (for eventual analysis repetition), but differences in TAC concentration between hospital sampling and home-based self-sampling were lower than 10% [19]. A strong correlation between the reference wet blood method and microsampling TAC determination was demonstrated in a study by Tron et al. [29]. More than 90% of the differences evaluated by the Bland–Altman plot were within the acceptable range [29]. Mathew [31] performed simultaneous assays of tacrolimus and creatine concentrations in VAMS and concluded that Mitra™ is a better option than DBS for renal function monitoring after transplantation. Veenhof et al. presented three different factors in their study, which influenced the higher systematic bias, i.e., anticoagulant impact, extraction solvent, and/or invisible under sampling [28].

## 3. Around VAMS—Strengths, Weaknesses, and Relevant Aspects

This section describes selected problems, points of attention, and propositions for solving frequent analytical and preanalytical difficulties. A summary of our proposal for the issue evaluation is presented in Table 4.

### 3.1. Hematocrit Effect

Haematocrit is the percentage ratio of RBC in the blood volume. Usually, this parameter ranged between 40–50% and 35–45% for adult men and women. In neonates, the HCT level may increase in the first days of life and oscillate to 65% of RBC in whole blood volume [24,25]. Factors influencing HCT level were sex, age, comorbidities, ethnicity, and polypharmacotherapy. In particular, the hematocrit level directly represented blood viscosity, and the density–variability of these parameters significantly influenced the sample quality and analyte recovery from the microsampling device (tip or spot). Volumetric microsampling (including volumetric methods in common with DBS) appears to be an alternative to the HCT effect. However, the manufacturer of the Mitra™ device reported that the HCT level did not influence sample recovery, and some studies have evaluated this effect during validation. Some approaches have been introduced to evaluate and predict the HCT effect in the case of DBS and VAMS. Correction with potassium level is considered a marker for HCT prediction in DBS and VAMS. HCT may play a vital role in some analytes, especially those with high RDB-binding sites, such as TAC and CSA [25].

In both studies, Paniagua-Gonzales et al. [23] evaluated that the HCT effect has not been statistically considered, especially during the determination of TAC and MPA. In a study by Scuderi et al. [30], correction factors according to HCT were introduced into formulas for calculating MPA and PRE concentrations (as drugs typically analyzed in the plasma). In our study about TAC in the pediatric population, no influence of hematocrit for analyte recovery from VAMS has been evaluated [18].

In the Kita [20,21] study, the impact of HCT on accuracy was examined at three different levels (0.20, 0.45, and 0.65) according to the drug model with high blood partition (TAC). The extraction recovery values and the matrix effect fulfilled the acceptance validation criteria because all values were almost 100% (for both LQC, low quality control, and HQC, high quality control). These studies confirmed that VAMS might be used for TAC determination in samples with different HCT levels, but further evaluation of other drugs should include other chemical properties.

In the study with everolimus performed in 2018, the biases for low and high HCT values for LQC, MQC (medium quality control), and HQC were diametrically different, ranging from −20 to +31% [33]. The author hypothesized that a large amount of RBS may chopped analytes in the VAMS tip pores, influencing extraction recovery. It has been proven that the HCT effect and its influence on extraction should be evaluated in every validation process. In a Norwegian study conducted in 2019 by Vethe et al. [19], water was used as the extraction medium. The recovery from the tip was satisfactory, but the effect of the HCT was not tested.

Tron et al. [29] evaluated the influence of HCT on TAC determination in VAMS samples. No significant effect on TAC concentration was observed in the standard HCT level (0.40) or in the more extensive range of 0.20–0.60 (2.5–30 ng/mL concentration). Koster et al. [22] concluded that biases caused by the HCT effect were within the acceptance range (<15%) for all analytes except CSA (HCT levels 0.20–0.60 and 0.27–0.60, respectively). For HQC, in common with lower HCT levels, recoveries were reduced for mTOR kinase inhibitors.

### 3.2. Automatization of the VAMS Methods

Many TDM laboratories use automation to increase proficiency, efficiency, and repeatability. Currently, DBS card platforms perform automated spot recognition, punching, and sample extraction. For newborn microsampling techniques such as VAMS, automatization is still required. Only in the study performed by Broek et al., the semi-automated approach during analytical processing has been used. The introduction of VAMS for routine medical care should begin with studies on fully or partially automated protocols for this technique in TDM laboratories. During TDM of drugs, many samples should be collected; therefore, automation of the analytical process is required [36]. The VAMS sampler was designed to be compatible with classic, popular automatic liquid handling systems for potential automatization of sample preparation.

### 3.3. Sampling in the Home–Point-of-Care (POC) as a Method for Adherence Improvement

**Figure 2:**
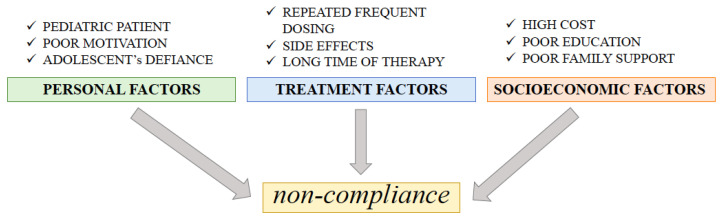
The main factors causing noncompliance in transplant patients [37].

The European Society for Patient Adherence, Compliance, and Persistence defined adherence as “the process by which the patients take their medication as prescribed” [37]. Noncompliance with a therapeutic regimen is a complex problem, especially for adolescent and child transplant recipients. A study by Blowey et al. [38] on compliance evaluated by monitoring CSA levels, attending clinic visits, individual interviews, and unexplained late graft dysfunction identified noncompliance as the main factor in late graft loss, accounting for 71% of cases. In a recent study, Rianthavorn et al. [39] noted that that “the long-term transplant outcome in adolescents is disappointing despite the best 1-year graft survival. Non-adherence with immunosuppressive medications is one of the most significant contributing factors for graft rejection and loss in adolescents.” The primary risk of non-adherence may result in low-income family support and the child’s psychological functioning. Other reasons that may cause noncompliance in the pediatric population are shown in Figure 2 [37]. Life-long immunosuppressive therapy is necessary for each patient after transplantation to avoid chronic or acute rejection episodes. Higher immunosuppressant concentration variability (in the case of non-adherence) is associated with acute rejection, decreased graft survival, and an increased cost of therapy. Home-based microsampling (especially VAMS) is an increasingly promising solution to problems associated with adherence to immunosuppressive therapy [37,38,39].

### 3.4. Proficiency Testing as a Method for Global Standardization

To date, only one multicenter study on proficiency testing was performed by Veenhof in 2017–2019 [35]. Fourteen TDM laboratories from seven countries participated in the pilot test for monitoring five immunosuppressants with microsampling techniques. TAC, CSA, EVE, SIR, and MPA concentrations were tested with LC-MS/MS using the following microsampling devices: DBS (Whatman 903 and DMPK-C) and VAMS (HemaXis™, Mitra™, and Capitainer-B™). In eight laboratories, nonvolumetric DBS was the leading microsampling device; in four participants, the Mitra™ device was used, and one was used for each of the last techniques, namely HemaXis™ and Capitainer-B™. Seven laboratories participated in proficiency testing, joined scarcely in the third round [35]. Therefore, some results should be interpreted carefully. All included laboratories analyzed TAC, eight performed assays for CSA determination, and seven and six of laboratories analyzed EVE and SIR, respectively, whereas only two participants quantified MPA. Fifty percent of the participating laboratories used ASC as the IS, but the validation results were statistically similar to those of the methods with ^13^C,D_2_-TAC for TAC determination [35].

The extraction procedure is the main factor potentially influencing the high inter-laboratory variability. Eight and six laboratories used ZnSO_4_ and acetonitrile for sample purification. The high variability of centrifuging and/or vortexing parameters may cause relative differences in proficiency testing results. Half of the laboratories performed clinical validation of their studies, whereas nine routinely used microsampling methods in daily patient healthcare. Finally, Veenhof et al. concluded that harmonization and standardization of the microsampling devices used in the TDM of immunosuppressants are necessary because of the high interlaboratory variability compared to wet blood methods. The main limitation of proficiency testing is that a small number of laboratories participated in the study because the study was performed in 2017–2019 [35]. SIR, EVE, and MPA were analyzed only in the last testing round; therefore, these drug quantifications’ results should be interpreted carefully. The main limitation of MPA determination using a microsampling device is the evaluation of the correlation between its values in blood and serum; in that case, standardization of the mathematical formula is also necessary [9,35]. The authors pointed out that global harmonization according to microsampling devices is a potential solution to the problem of high cross-laboratory variability in the results of proficiency testing. It also needs to be clarified that proficiency testing is obligatory for all medical laboratories and microsampling cases, according to International Organization for Standardization (ISO) rules [9,33,40].

### 3.5. Future Perspective on VAMS for Immunosuppressants TDM

The issues associated with traditional sampling and BDS were overcome by introducing novel microsampling methods. Following a questionnaire study performed by Bioanalysis-zone.com, 29% of the respondents were interested in microsampling in 2014, whereas 49% of the included laboratories started microsampling in 2016. In every case, more than half of the respondents used microsampling to monitor small molecules, including TDM [41]. Based on PubMed [42], 126 studies with significant annual growth have been described according to volumetric microsampling. Twenty of these publications have been concerned wholly or partly with the utility of immunosuppressive therapy [42]. Preferentially for immunosuppressive therapy, microsampling enables medical doctors and pharmacists to obtain enough biological samples within each drug dosing interval range without specific requirements according to the sampling time. Recently, Mathew et al. [31] reported the successful use of Mitra™ for monitoring TAC and creatinine levels in the Indian population. However, the results obtained from the BDS and VAMS samples were equivalent, but the patients who participated in the study indicated that the Mitra™ device was a better alternative for the self-sampling of blood samples. It seems that interest on the part of patients in the VAMS technique brings a brighter future for microsampling in immunosuppressive pharmacotherapy. The development of microsampling may result in the three branches of the immunosuppressant tract, namely, their components in whole blood, intracellular concentrations, and pharmacodynamic (PD) biomarkers [9,10,43]. Although good clinical results for patients have been observed, more detailed regulatory feedback from medical agencies is still needed, as well as multicenter studies on the clinical application of VAMS. On the other hand, home-based sampling and, consequently, better adherence to therapy can benefit long-term economic aspects. It is estimated that owing to the clear benefits of using VAMS, the utility of this technique and immunosuppressant monitoring will grow annually [9,13].

Gustavsen et al. [44] more extensively used Mitra™ as a valuable tool for venous TAC AUC prediction, using a limited sample strategy (LSS for AUC_0–12_ prediction). The authors pointed out that the study has some limitations in conclusions (such as lack of long-term PK profiles after Tx, variability of TAC absorption, etc.), but constitutes a great utility of microsampling according to population PK. The strict benefit is the possibility of frequent and easy-to-perform sampling in patients’ home conditions [44,45]. To date, only Kindem et al. [46] have prescribed the utility of VAMS for TAC determination in pediatric transplant patients. This is proof that future perspectives the subsequent studies are greatly needed because of the indisputable advantages of microsampling for young patients.

## 4. Other Volumetric Microsampling Methods

A few critical points should be reviewed to evaluate the usefulness of each microsampling method. First, the sampling process should be easy for patients without complicated steps. Secondly, the unit price of the device, as well as the accompanying costs of sampling, transport, storage, and analyses, should be profitable with its usefulness and clinical application. Finally, the analytical process should be compatible with typical diagnostics laboratories, including sample preparation, analytical method, and automatization [9,13].

Microsampling methods are divided into volumetric and nonvolumetric techniques. In TDM of immunosuppressants, the appropriate volume of select body fluid is critical for correct dosage adjustment and improvement of therapy from a long-term perspective. Generally, volumetric methods are a better choice for the TDM of narrow-therapeutic index drugs (NTIDS) because of the potential low variability of sample volume, better homogeneity, and sampling automatization. However, the usefulness of each device (regardless of type) should be checked according to the target population, analytes, type of matrix, laboratory proficiency, coordination reasons, and clinical outcome.

In the following sections, a short characteristic of the most popular microsampling methods, in contrast to the VAMS-Mitra™ device, is described. A summary of the selected microsampling methods is provided in Table 5.

### 4.1. Capillary Microsampling (CMS)

Using capillary microsampling (CMS), a small volume of whole blood was collected. Recently, the most popular devices are Drummond™ (Drummond Scientific Company, Broomall, PA) and Vitrex™ (Vitrex Medical A/S, Herlev, Denmark) capillary tubes. Both manufacturers produced several types of devices, namely glass or plastic capillaries, that were plain or coated with various anticoagulant agents, such as sodium, ammonium, lithium heparin, or EDTA. The blood is filled by capillary action into a narrow tube and may be closed by a vax on either side of the capillary. Generally, CMS is nonvolumetric; however, unique blood collection can be performed using special volume-calibrated tubes. Variable volumes of capillaries (0.5–100 μL) are available on the diagnostic market, and one of the manufacturers declared a ±0.50% accuracy bias and variability at 0.60–0.75% level [47,48].

Additionally, even blood samples should be stored in the same conditions as in the case of classic venipuncture samples, but on the other hand, DBS cards may be used to improve sample stability. Therefore, CMS tubes cannot be transported or delivered by mail at room temperature. Using CMS, sampling is easy but should be performed with medical staff supervision. In contrast to dried blood techniques, the hematocrit effect on the sample volume and analyte recovery was insignificant. Automation of CMS sample analysis is particularly limited because of the difficulties associated with sample preparation. The fragility of tubes is a key factor responsible for their limited stability and the necessity for special conditions during transport. Capillary blood can be collected from the earlobe, forearm, heel, palm, fingertip, or arm; however, the fingerprick with a lancet is the most often used method for sample collection [11,47,48].

To date, no study has focused on CMS during the TDM of immunosuppressants.

### 4.2. Dried Blood Spots (DBS)

Mention in Section 1. DBS is the most well-known microsampling method developed in 1961 by Guthrie for screening investigations according to phenylketonuria in neonates [10,13,14]. The basis of the DBS technique is a cellulose paper card (the most popular is Whatman or Ahlstrom) with punch points, where the blood samples may be loaded by a capillary glass tube after the finger, toe, or heel prick. This technique is the best-known microsampling method for home-based self-sampling. The most difficult problems with DBS method validation are focused on recovery, matrix effect, process efficiency, volume effect, hematocrit effect, sample homogeneity, and volcano effect [10]. Cellulose cards (modified or non-modified) are used for DBS, such as Whatman 903, Ahlstrom 226, Whatman FTA, FTA Elute, DMPK-A, and DMPK-B. Complex matrices, such as whole blood, are complicated in the case of DBS, and chromatographic stratification on a cellulose card is often observed. As in the case of all microsampling devices, sample storage, delivery, and preparation are simplified. The extraction conditions and solvents used were similar to those used recently in methods based on VAMS. Generally, internal standards (ISs) are added to the DBS card incorporation before sampling and extraction. Other approaches imply that IS may be added to the blood matrix before spotting the card, directly with the extraction solvent, or before the sample cleaning up (purification). The ideal method is to incorporate a DBS card before sampling to reduce the matrix effect and increase analyte recovery maximally. Because of the small sample volume and relatively high dilution during extraction, more sensitive and selective methods should be used for DBS sample quantification [10,11,13,14].

DBS is the most popular microsampling method, and numerous analytical methods have been developed to determine immunosuppressants. Additionally, the IATDMCT guided DBS usage in the TDM of various NTIDs [10].

### 4.3. Capitainer^TM^

**Figure 3:**
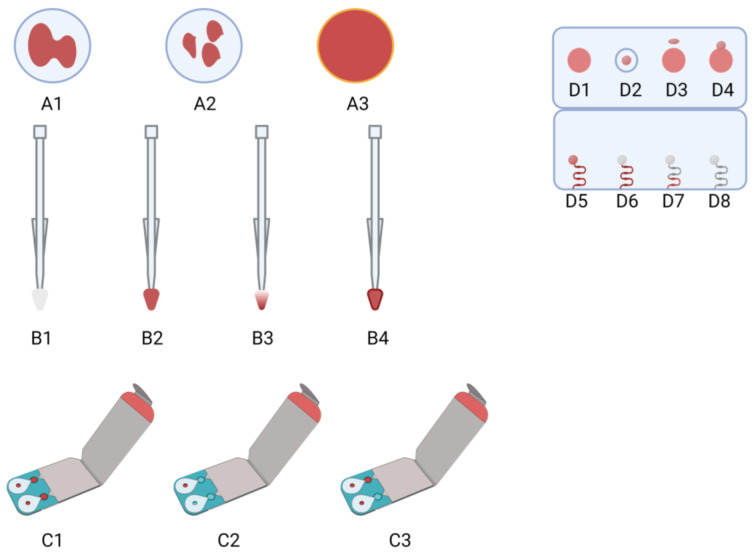
Comparison of typical problems during sampling for selected microsampling methods: (A) DBS, (B) VAMS—Mitra™, (C) qDBS—Capitainer™, and (D) Hemaxis™. Issues description: (**A1**) layering; (**A2**) multiple blood applications; (**A3**) chromatographic effect (serum ring); (**B1**) blank VAMS sampler; (**B2**) correctly loaded VAMS sampler; (**B3**) unloaded VAMS sampled; (**B4**) overloaded VAMS sampler; (**C1**) correctly sampled qDBS; (**C2**) unloaded qDBS; (**C3**) correctly loaded and unloaded sampler windows (left and right, respectively); (**D1**,**D3**,**D5**) sample correctly loaded and punched; (**D2**,**D4**,**D6**,**D7**) unloaded blood sample and/or potential punch mistakes; (**D8**) an open capillary system for comparison purposes. Created with BioRender.com under publishing rights.

The qDBS Capitainer™ microsampling device (Capitainer AB, Solna, Sweden) is a valuable tool based on the quantitative enabling of two fixed volumes of capillary blood simultaneously with the same sampler. This device contains two windows (ports) in common with two separate capillary channels, which obtained 18μL of blood volume and collected 10μL onto Ahlstrom 270 paper with a precision of <5%. As reported by Capitainer, the qDBS solution eliminates the overfilling risk and underfilling, and the color indication provides sampling success [50]. After sampling, the device is dried under ambient conditions for a minimum of two hours, after which drops are carefully removed and prepared for extraction. The validated operating temperature range oscillated between +15 °C and +35 °C, whereas the hematocrit range was established as 0.25–0.55. The other analytical steps were similar to those of other microsampling strategies. Exampled errors according to Capitainer™ are presented in Figure 3. Similar to other microsampling devices, the sampling process may be performed by almost anyone. The quality of the sampler design directed for classic mail delivery allows for the preservation of satisfactory sample stability. When following the manufacturers’ instructions: open, prick, apply, and post [51].

Additionally, studies have reported no influence of the hematocrit level or lipids on sample quality. Velghe and Stove [49,50] tested the potential influence of the obtained blood drop and hematocrit levels on the final analyte concentration. This is the only study that has experimentally described the possibility of Capitainer-B using TDM to overcome the HCT effect and sample volume variability. No studies have focused on using this device to monitor immunosuppressant concentrations. In a pilot proficiency testing scheme, only one of participating laboratories use Capitainer-B™ as a device for blood collection during TAC, SIR, EVE, and CSA concentration monitoring [35].

### 4.4. Hemaxis^TM^

HemaXis™ is an innovative microsampling device based on the hybrid common DBS technique with volumetric microsampling, introduced by DBS System SA, Gland, Switzerland. This device’s signature strengths are four DBS per device and high-quality 903 Protein Saver filter card grade (standard cassette format). The volume which may be obtained with one DBS oscillates in: 10.0 ± 0.5μL (with a 95% confidence interval). The HemaXis™ blood collection was based on a fixed volume of blood obtained using a fingerprick [51]. After absorption, the sampler was covered, and the blood sample was directly transferred onto a cellulose filter card without additional manual steps or special equipment. However, the manufacturer described the high quality of sampling. Delahaye et al. pointed out that the HemaXis™ device is characterized by a relatively higher risk of external contamination. In pilot proficiency testing, only one laboratory performed TAC, EVE, and MPA determinations using the HemaXis™ device [51]. It is necessary to note that using microsampling devices for MPA monitoring is problematic because it is essential to correlate drug exposure in whole blood and plasma levels. More precisely, MPA strongly binds into serum protein, and classic analytical methods are based on its determination in plasma. On the other hand, by microsampling devices, whole capillary blood is collected. Therefore, the mathematical correlation formula between results obtained from different matrices is strictly necessary in pharmacokinetic studies [35]. HemaXis™ DB is an FDA Class 1 medical device; therefore, it may be used only for development and research studies and not for medical diagnostics. Exampled errors according to HemaXis™ are presented in Figure 3. To date, only the Zwart et al. study has focused partly on the HemaXis™ microsampling device [27].

### 4.5. HemaPen^TM^

Trajan Scientific and Medical (Melbourne, VA, Australia) developed a semi-automated microsampling HemaPen™ device containing four capillaries with four paper discs. This system was coated with EDTA and absorbed a fixed blood volume of 2.74 μL of capillary blood in each case. The main advantage is that four HemaPen™ samples were collected from the same source (the 20 μL blood drop is recommended) [52]. As well as in the case of other microsampling devices, HemaPen™ seems to be an alternative strategy for minimalizing HCT and complex storage conditions limited. HemaPen™ is supplied for therapeutic or IVD use in Australia, New Zealand, the UK, the EU, and the USA [52]. This device is supplied outside the territories listed above for research purposes only, not for therapeutic or diagnostic use. To date, no study has reported on the use of this device for immunosuppressant monitoring. Recently, this device’s main applications have focused on immunoglobulins and immunological biomarkers [9,52].

## 5. Application of Microsampling for Other Matrices Collecting

Microsampling is a universal collection technique of matrices other than whole (capillary) blood. As mentioned above, the best matrix for MPA determination is plasma, but in the case of Mitra™, the self-collection of plasma is the most difficult. DBS technique modifications, known as dry plasma spot (DPS) or dry saliva spot (DSS), are viable alternatives for achieving higher sample stability. However, the manufacturer and some studies declared that Mitra™ is ready to use in case of other matrices samplings, such as urine, saliva, and cerebrospinal fluid, but in the therapeutic drug monitoring of immunosuppressants, the matrices do not matter much [9,11,13].

## 6. Conclusions

Volumetric absorptive microsampling (VAMS) is an alternative tool for blood sampling, transport, and storage. It could be expected that for a broad spectrum of biochemical analyses, including TDM, VAMS successfully replaced the standard sampling venipuncture technique, omitting the blood collection facility, significantly reducing blood volume, and finally being much more friendly for the patient. Combining VAMS with LC-MS/MS seems to be a promising analytical method for pharmacokinetic analyses and TDM. Another innovative issue is using alternative tools for non-adherence monitoring, which is characteristic of pediatric transplant recipients. The VAMS chromatographic method for simultaneous monitoring of TAC and MPA, in common with biochemical parameters (e.g., creatinine and eGFR) and evaluation of adherence, may improve immunosuppressive therapy and consequently extend graft survival.

Finally, the unit price of one Mitra™ sampler is higher than that of the DBS card or venipuncture kit; however, microsampling has more advantages and benefits for future costs of life-long immunosuppressive therapy.

This technique is patient-friendly and simplifies clinical trials and drug monitoring. This provides a better experience for vulnerable patients, especially children. Following the manufacturers’ instructions, blood collection can be performed anywhere, at any time, and by almost anyone. These techniques are becoming increasingly popular, with an annual global growth rate, and the SARS-CoV-2 pandemic has increased the need for POP devices, including self-sampling devices [43]. The Theranos™ dire case showed how novel approaches for diagnostics are needed and demanded; however, they should be carefully and thoroughly examined and evaluated for clinical application. Microsampling techniques must become increasingly important in clinical trials and home-based monitoring of drugs, illnesses, or systemic organ functioning. In conclusion, we believe that microsampling devices are a chance to improve the quality of immunosuppressive therapy.

## Figures and Tables

**Table 3:** Summary of analytical characteristics of immunosuppressive drug assays in common with microsampling techniques.

Drug Name	Analytical Method	Injection Volume	Selected Chromatographic Conditions	Internal Standard	Selected Apparatus Conditions	Calibration Range (Linearity)	Reference
CSA	(+)ESI-LC-MS/MS	20 μL	C_18_ chromatographic column, mobile phase: ammonium formate buffer pH 3.5 and methanol, flow rate: 1 mL/min, gradient flow	D_12_-CSA	RF lens = 93 V CE = 15 eV positive mode (ammonium adduct monitoring)	10–500 ng/mL	[22]
	(+)ESI-LC-MS/MS and US-LC-MS/MS	20 μL	C_18_ chromatographic column, mobile phase: ammonium formate and formic acid in water and acetonitrile flow rate: 0.5 mL/min, gradient flow	D_4_-CSA	CV = 20 V CE = 19 eV positive mode (ammonium adduct monitoring)	20–2000 ng/mL	[23]
	(+)ESI-LC-MS/MS	35 μL	C_18_ chromatographic column, mobile phase: n.d., flow rate: n.d. gradient flow	D_12_-CSA	RF lens = n.d. CE = n.d. positive mode (ammonium adduct monitoring)	22.7–937.0 ng/mL	[26]
	(+)ESI-LC-MS/MS	40 μL	C_18_ chromatographic column, mobile phase: formic acid, ammonium in water and methanol, flow rate: 0.45 mL/min, gradient flow	D_12_-CSA	RF lens = n.d. CE = n.d. positive mode (ammonium adduct monitoring)	0–1904 μg/L	[27]
TAC	(+)ESI-LC-MS/MS	20 μL	C_8_ chromatographic column, mobile phase: water with formic acid and ammonium acetate and methanol with formic acid ammonium acetate., flow rate: 0.60mL/min, gradient flow	^13^C,D_2_-TAC	RF lens = 82 V CE = 23 eV positive mode (ammonium adduct monitoring)	1.3–60 μg/L	[19]
	(+)ESI-LC-MS/MS	3 μL	C_18_ chromatographic column, mobile phase: water, and methanol (with acetic acid), flow rate: 0.25 mL/min, gradient flow	ASC	RF lens = 60 V CE = 40 eV positive mode	1–250 ng/mL	[20]
	(+)ESI-LC-MS/MS	3 μL	C_18_ chromatographic column, mobile phase: water and methanol (with ammonium acetate and acetic acid) flow rate: 0.35 mL/min, gradient flow	ASC	RF lens = 40 V CE = 27 eV positive mode (ammonium adduct monitoring)	0.2–250 ng/mL	[21]
	(+)ESI-LC-MS/MS	20 μL	C_18_ chromatographic column, mobile phase: ammonium formate buffer pH 3.5 and methanol, flow rate: 1 mL/min, gradient flow	^13^C,D_2_-TAC	RF lens = 82 V CE = 20 eV positive mode (ammonium adduct monitoring)	1–50 μg/L	[22]
	(+)ESI-LC-MS/MS and US-LC-MS/MS	20 μL	C_18_ chromatographic column, mobile phase: ammonium formate and formic acid in water and acetonitrile flow rate: 0.5 mL/min, gradient flow	^13^C,D_2_-TAC	CV = 22 V CE = 20 eV positive mode (ammonium adduct monitoring)	0.5–50 ng/mL	[23]
	(+)ESI-LC-MS/MS	35 μL	C_18_ chromatographic column, mobile phase: n.d., flow rate: n.d. gradient flow	^13^C,D_2_-TAC	RF lens = n.d. CE = n.d. positive mode (ammonium adduct monitoring)	2.20–41.30 ng/mL	[26]
	(+)ESI-LC-MS/MS	40 μL	C_18_ chromatographic column, mobile phase: formic acid, ammonium in water and methanol, flow rate: 0.45 mL/min, gradient flow	^13^C,D_2_-TAC	RF lens = n.d. CE = n.d. positive mode (ammonium adduct monitoring)	2.18–42.4 μg/L	[27]
	(+)ESI-LC-MS/MS	20 μL	C_18_ chromatographic column, mobile phase: ammonium formate buffer pH 3.5 and methanol, flow rate: 1 mL/min, gradient flow	^13^C,D_2_-TAC	RF lens = 82 V CE = 20 eV positive mode (ammonium adduct monitoring)	1–50 μg/L	[28]
	(+)ESI-LC-MS/MS	10 μL	C_18_ chromatographic column, mobile phase: 95% acetonitrile and 5% 10 mM ammonium acetate in water, flow rate: 0.1–0.6 mL/min, isocratic flow	ASC	RF lens = 135 V CE = 20 eV positive mode (ammonium adduct monitoring)	2.25–42.9 ng/mL	[29]
	(+)ESI-LC-MS/MS	n.d.	C_18_ chromatographic column, mobile phase: ammonium acetate with formic acid in water and methanol, flow rate: 0.4 mL/min, gradient flow	^13^C,D_2_-TAC	RF lens = n.d. CE = n.d. positive mode (ammonium adduct monitoring)	0–40 μg/L	[30]
	(+)ESI-LC-MS/MS	10 μL	C_18_ chromatographic column, mobile phase: water with ammonium fluoride and formic acid, and methanol: acetonitrile with ammonium fluoride and formic acid, flow rate: 0.75 mL/min, gradient flow	ASC	CV = n.d. CE = 22 eV positive mode (ammonium adduct monitoring)	0–60 μg/L	[18]
	(+)ESI-LC-MS/MS	20 μL	C_18_ chromatographic column, mobile phase: ammonium acetate buffer with 0.1% formic acid and ammonium acetate in methanol with 0.1% formic acid, flow rate: 0.4 mL/min, gradient flow	ASC	CV = 27 V CE = 20 eV positive mode (ammonium adduct monitoring)	1.45–29.28 μg/L	[31]
	(+)ESI-LC-MS/MS	50 μL	C_18_ chromatographic column, mobile phase: formic acid, ammonium in water and methanol, flow rate: 0.45 mL/min, gradient flow	ASC	RF lens = 35 V CE = n.d. positive mode (ammonium adduct monitoring)	2.23–84 μg/L	[32]
MPA	(+)ESI-LC-MS/MS	20 μL	C_18_ chromatographic column, mobile phase: 20 ammonium formate buffer pH 3.5 and methanol, flow rate: 1 mL/min, gradient flow	^13^C,D_3_-MPA	RF lens = 58 V CE = 22 eV positive mode (ammonium adduct monitoring)	100–1500 ng/mL	[22]
	(+)ESI-LC-MS/MS and US-LC-MS/MS	20 μL	C_18_ chromatographic column, mobile phase: ammonium formate and formic acid in water and acetonitrile flow rate: 0.5 mL/min, gradient flow	^13^C,D_3_-MPA	CV = 30 V CE = 15 eV positive mode (ammonium adduct monitoring)	75–7500 ng/mL	[23]
	(+)ESI-LC-MS/MS	10 μL	C_18_ chromatographic column, mobile phase: formic acid, ammonium in water and methanol, flow rate: 0.45 mL/min, gradient flow	^13^C,D_3_-MPA	RF lens = n.d. CE = n.d. positive mode (ammonium adduct monitoring)	0.5–20 mg/L	[27]
	(+)ESI-LC-MS/MS	n.d.	C_18_ chromatographic column, mobile phase: ammonium acetate with formic acid in water and methanol, flow rate: 0.4 mL/min, gradient flow	^13^C,D_3_-MPA	RF lens = n.d. CE = n.d. positive mode (ammonium adduct monitoring)	0–20 mg/L	[30]
	(+)ESI-LC-MS/MS	50 μL	C_18_ chromatographic column, mobile phase: formic acid, ammonium in water and methanol, flow rate: 0.45 mL/min, gradient flow	^13^C,D_3_-MPA	RF lens = 35 V CE = n.d. positive mode (ammonium adduct monitoring)	0–20 mg/L	[32]
EVE	(+)ESI-LC-MS/MS	20 μL	C_18_ chromatographic column, mobile phase: ammonium formate buffer pH 3.5 and methanol, flow rate: 1 mL/min, gradient flow	^13^C_2_,D_4_-EVE	RF lens = 88 V CE = 16 eV positive mode (ammonium adduct monitoring)	1–50 μg/L	[22]
	(+)ESI-LC-MS/MS and US-LC-MS/MS	20 μL	C_18_ chromatographic column, mobile phase: ammonium formate and formic acid in water and acetonitrile flow rate: 0.5 mL/min, gradient flow	^13^C_2_,D_4_-EVE	CV = 20 V CE = 16 eV positive mode (ammonium adduct monitoring)	0.5–50 ng/mL	[23]
	(+)ESI-LC-MS/MS	35 μL	C_18_ chromatographic column, mobile phase: n.d., flow rate: n.d. gradient flow	^13^C_2_,D_4_-EVE	RF lens = n.d. CE = n.d. positive mode (ammonium adduct monitoring)	2.3–44.2 ng/mL	[26]
	(+)ESI-LC-MS/MS	40 μL	C_18_ chromatographic column, mobile phase: formic acid, ammonium in water and methanol, flow rate: 0.45 mL/min, gradient flow	^13^C_2_,D_4_-EVE	RF lens = n.d. CE = n.d. positive mode (ammonium adduct monitoring)	0–41.6 μg/L	[27]
	(+)ESI-LC-MS/MS	5 μL	C_18_ chromatographic column, mobile phase: 20 mM ammonium formate in water and methanol flow rate: 0.4 mL/min, gradient flow	^13^C_2_,D_4_-EVE	RF lens = n.d. CE = 30 eV positive mode (ammonium adduct monitoring)	2.5–100 μg/L	[33]
SIR	(+)ESI-LC-MS/MS	20 μL	C_18_ chromatographic column, mobile phase: ammonium formate buffer pH 3.5 and methanol, flow rate: 1 mL/min, gradient flow	temsirolimus	RF lens = 83 V CE = 15 eV positive mode (ammonium adduct monitoring)	1–50 μg/L	[22]
	(+)ESI-LC-MS/MS and US-LC-MS/MS	20 μL	C_18_ chromatographic column, mobile phase: ammonium formate and formic acid in water and acetonitrile flow rate: 0.5 mL/min, gradient flow	^13^C_2_,D_4_-EVE	CV = 22 V CE = 16 eV positive mode (ammonium adduct monitoring)	0.5–50 ng/mL	[23]
	(+)ESI-LC-MS/MS	35 μL	C_18_ chromatographic column, mobile phase: n.d., flow rate: n.d. gradient flow	^13^C,D_3_-SIR	RF lens = n.d. CE = n.d. positive mode (ammonium adduct monitoring)	2.20–47.20 ng/mL	[26]
	(+)ESI-LC-MS/MS	40 μL	C_18_ chromatographic column, mobile phase: formic acid, ammonium in water and methanol, flow rate: 0.45 mL/min, gradient flow	^13^C,D_3_-SIR	RF lens = n.d. CE = n.d. positive mode (ammonium adduct monitoring)	0–47 μg/L	[27]
	(+)ESI-LC-MS/MS	10 μL	C_18_ chromatographic column, mobile phase: 0.1% formic acid and methanol, flow rate: 0.6 mL/min, gradient flow	^13^C,D_3_-SIR	RF lens = 100 V CE = 58 eV positive mode (ammonium adduct monitoring)	1–250 ng/mL	[34]

CSA—cyclosporine, TAC—tacrolimus, MPA—mycophenolic acid, EVE—everolimus, SIR—sirolimus, n.d.—no data available in the study, (+)ESI-LC-MS/MS—liquid chromatography-tandem mass spectrometry in positive ion mode, US-LC-MS/MS—liquid chromatography-tandem mass spectrometry with unispray, RF lens—radio frequency lens voltages, CE—collision energy, CV—cone voltage, D_4_-CSA/D_12_-CSA—deuterated cyclosporin, ASC—ascomycin, ^13^C,D_2_-TAC—deuterated tacrolimus, ^13^C,D_3_-MPA—deuterated mycophenolic acid, ^13^C_2_,D_4_-EVE—deuterated everolimus, ^13^C,D_3_-SIR—deuterated sirolimus.

**Table 4:** Potential solutions for selected analytical and preanalytical aspects of microsampling.

Difficulty	Workable Solutions
hematocrit effect	Testing hematocrit effect in case of validation and every method modification and introducing correction formula based on, e.g., potassium level **and/or** Modification attempts in extraction parameters **and/or** Monitoring of self-sampling correctness with a simple questionnaire **and/or** Monitoring of hematocrit levels according to drug concentrations regularly
mistakes in self-sampling	Regular revision of sampling training for patients **and/or** Good availability of explicit sampling instruction for patients **and/or** Additional resources, i.e., as tutorial videos for patients **and/or** Monitoring of self-sampling correctness with a simple questionnaire **and/or** Responsible guardians/parents care during sample collection (in the case of pediatric patients)
limited sample stability	Limited whole blood dilution through low volumes of calibrators and other solutions addition (particularly <5% of sample volume) **and/or** Clear guidelines about sample storage and preparing to send for patients (according to desiccant and drying) **and/or** Modification of method protocol—controlling every step according to influence for sample stability (During validation, according to EMA/FDA guidelines)
IS incorporation step	Impregnation of the sampler with IS before sample collecting **or** Spiking the samples before or after the extraction process **or/and** The two-stage approach according to liquid–liquid extraction **or/and** Changing of internal standard (another structural analog or isotope-stable internal standard)
drying conditions (time, temperature, and humidity)	Testing selected parameters during method development (in-vitro conditions) **and/or** Drying and sample storage in controlled conditions **and/or** Clear guidelines about sample storage and preparing to send for patients (according to desiccant and drying)
sample reanalysis necessity	Collecting a few samples at the same time (Simultaneously, replicate samples) **and/or** Collection of the higher volume of whole blood prior to microsampling
sampler contamination	In-vitro validation according to potential chemical contamination (creams, petroleum, etc.) **and/or** Appropriate disinfection of hands before fingerprick by patients **and/or** Using another microsampling method with limited contamination risk (i.e., HemaPen™) **and/or** Responsible guardians/parents care during sample collection (in the case of pediatric patients)
analytical method sensitivity	Optimization analytes recovery **and/or** Optimization sample purification and extraction protocol **and/or** Changing analytical method/apparatus/chromatographic column/detector conditions, etc. **and/or** The balance between sample injection volume and chromatographic parameters **and/or** Testing of method automatization

**Table 5:** Selected alternative microsampling techniques summarise the characteristics and compare them with the reference venipuncture method [11,13,15,47,48,49,50,51].

Feature	Venipuncture	CMS	DBS	VAMS Mitra™	VAMS HemaPen™	qDBS Capitainer™	VAMS/qDBS hemaXis™
Type of matrix	whole blood	capillary blood	capillary blood	capillary blood	capillary blood	capillary blood	capillary blood
Sampling	invasive (venipuncture)	noninvasive (fingerprick)	noninvasive (fingerprick)	noninvasive (fingerprick)	noninvasive (fingerprick)	noninvasive (fingerprick)	noninvasive (fingerprick)
Sample self-collection	impossible	impossible	possible (after training)	possible (after training)	possible (after training)	possible (after training)	possible (after training)
Sample volume	inaccurate (non-volumetric)	inaccurate (non-volumetric) or quantitative	inaccurate (non-volumetric)	quantitative (10, 20 or 30 μL) with RSD <4%	quantitative (2.74 μL)	quantitative (10 μL)	quantitative (10 μL)
Risk of sample contamination	possible (except vacuum and closed devices)	extremely high	high	high	extremely low	high	high
Visual control of blood loading	possible	possible	confined	possible	possible	possible (with control of sample volume)	possible (with control of sample volume)
Sample storage in RT	undesirable	undesirable	possible	possible, but with desiccant and in the dark	possible	possible, but with desiccant and in the dark	possible
Sample transportation	cold chain is required	cold chain is required	except for special conditions	except for special conditions	except for special conditions	except for special conditions	except for special conditions
Shipping by post	impossible	impossible	possible	possible	possible	possible	possible
Visualization	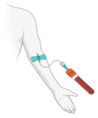	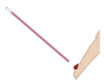	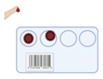	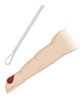	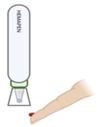	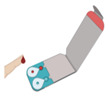	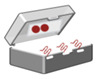

NVMS—nonvolumetric microsampling; qDBS—quantitative dried blood spot; VAMS—volumetric absorptive microsampling; DBS—dried blood spot; WB—whole blood collection with venipuncture. Visualisation was performed using BioRender.com under publishing rights.
